# Use of an endo fascial closure device for thoracoscopic patch repair of congenital diaphragmatic hernia

**DOI:** 10.3389/fped.2026.1919569

**Published:** 2026-09-14

**Authors:** Qianqing Li, Mingkun Liu

**Affiliations:** Department of Pediatric Surgery, Fujian Children's Hospital (Fujian Branch of Shanghai Children's Medical Center), College of Clinical Medicine for Obstetrics & Gynecology and Pediatrics, Fujian Medical University, Fuzhou, China

**Keywords:** congenital diaphragmatic hernia, neonate, new technical equipment, patch repair, thoracoscopy

## Abstract

**Purpose:**

This study describes a novel, simple, and reproducible technique for thoracoscopic patch repair of the diaphragm using an endo fascial closure device for the treatment of congenital diaphragmatic hernia (CDH).

**Methods:**

We retrospectively analyzed the clinical data of 18 patients with CDH who underwent thoracoscopic patch repair using the endo fascial closure device at our department between July 2021 and August 2026

**Results:**

In this study, follow-up of the 18 children ranged from 11 months to 4 years. The mean operative duration was 141.2 ± 55.4 min. The mean duration of ventilator support was 8.39 days, and the mean postoperative hospital stay was 17.4 days. No conversion occurred intraoperatively. Postoperative chest radiography and upper gastrointestinal contrast study confirmed normal positioning of the abdominal organs in all patients. One death occurred due to treatment abandonment, and the remaining 17 children recovered favorably.

**Conclusions:**

The use of an endo fascial closure device for diaphragmatic suture under thoracoscopy represents a simple, novel, and easily reproducible technique. This method may potentially improve suturing efficiency and facilitate the management of large diaphragmatic defects, although its advantages over conventional techniques require validation in comparative studies.

## Introduction

Congenital diaphragmatic hernia (CDH), resulting from congenital dysplasia of the posterolateral diaphragm, exerts varying impacts on cardiopulmonary function and overall patient condition, with severe cases posing life-threatening risks (1–3). In the era of minimally invasive surgery, thoracoscopic techniques have been increasingly adopted for the repair of congenital diaphragmatic defects (4, 5). However, current literature indicates that thoracoscopic repair is associated with notably higher recurrence rates (6). Acidosis during surgery is a considerable risk factor, and most reports focus on infants (6, 7). The prevailing view attributes these challenges to a steep learning curve, especially in neonatal cases where limited thoracic workspace and technical difficulties in patch placement compound procedural complexity (6, 8, 9).

To address these issues, this report introduces a novel and technically straightforward method: the application of an endo fascial closure device for diaphragmatic suturing during thoracoscopic patch repair of CDH. The key innovation of this technique lies in its simplicity—it repurposes a commonly available device to perform secure patch anchoring through a percutaneous approach, eliminating the need for complex intracorporeal suturing. This technique was designed to potentially enhance operative efficiency and reduce technical barriers by facilitating secure and expedient repair, even within the constrained anatomical confines characteristic of neonatal patients. The approach may represent a meaningful advance in minimally invasive pediatric surgery, warranting further evaluation in clinical practice.

## Materials and methods

This retrospective study analyzed consecutive cases of congenital diaphragmatic hernia (CDH) referred to our department at a national regional medical center between July 2021 and August 2026. All included patients were diagnosed based on clinical symptoms and comprehensive imaging examinations, including chest x-ray, computed tomography (CT), and upper gastrointestinal contrast studies, among other modalities. It is noteworthy that all patients in this series were referred from other hospitals or even from distant regions. As a result, fetal lung-to-head ratio (LHR) measurements had not been performed prenatally for any of the cases included in this study. Surgical intervention was performed strictly in accordance with standardized clinical indications as established in the literature (7). The indication for patch reinforcement was determined intraoperatively based on the morphological characteristics of the diaphragmatic defect. Patch repair was indicated when either of the following two conditions was present: (1) the defect was too large to allow primary closure without excessive tension, as judged by the surgeon; or (2) the residual native diaphragmatic rim was extremely thin, hypoplastic, and fragile, lacking sufficient structural integrity to provide reliable suture-holding strength for direct tension-free approximation.

During the study period, a total of 33 patients with CDH underwent repair at our institution. Among these, 15 were excluded: 4 underwent open repair, 2 underwent laparoscopic repair and 9 underwent thoracoscopic repair using conventional continuous suturing techniques without the device. The remaining 18 consecutive patients who met the inclusion criteria and received the endo fascial closure device were included in this technical case series. As this study was designed primarily to describe the feasibility and safety of a novel surgical technique, no concurrent matched control group was established for direct statistical comparison.

### Endo fascial closure device

The puncture tip is a sharp, hollow metal needle with a beveled opening, designed to pierce the skin and enter a body cavity. The shaft is a slender hollow tube through which a grasping forceps is passed internally. The operating end features a press-type spring mechanism. When the spring is in its naturally extended state, the grasping forceps is fully retracted inside the needle tip, allowing the needle to safely puncture tissues. When the spring is compressed, the miniature grasping forceps is pushed out of the needle tip. The forceps then closes and clamps the suture thread, after which the entire forceps body, along with the thread, retracts back into the lumen of the needle tip. It then passes through the tissue without obstruction and is ultimately brought out of the body (Figure 1).

**Figure 1:**
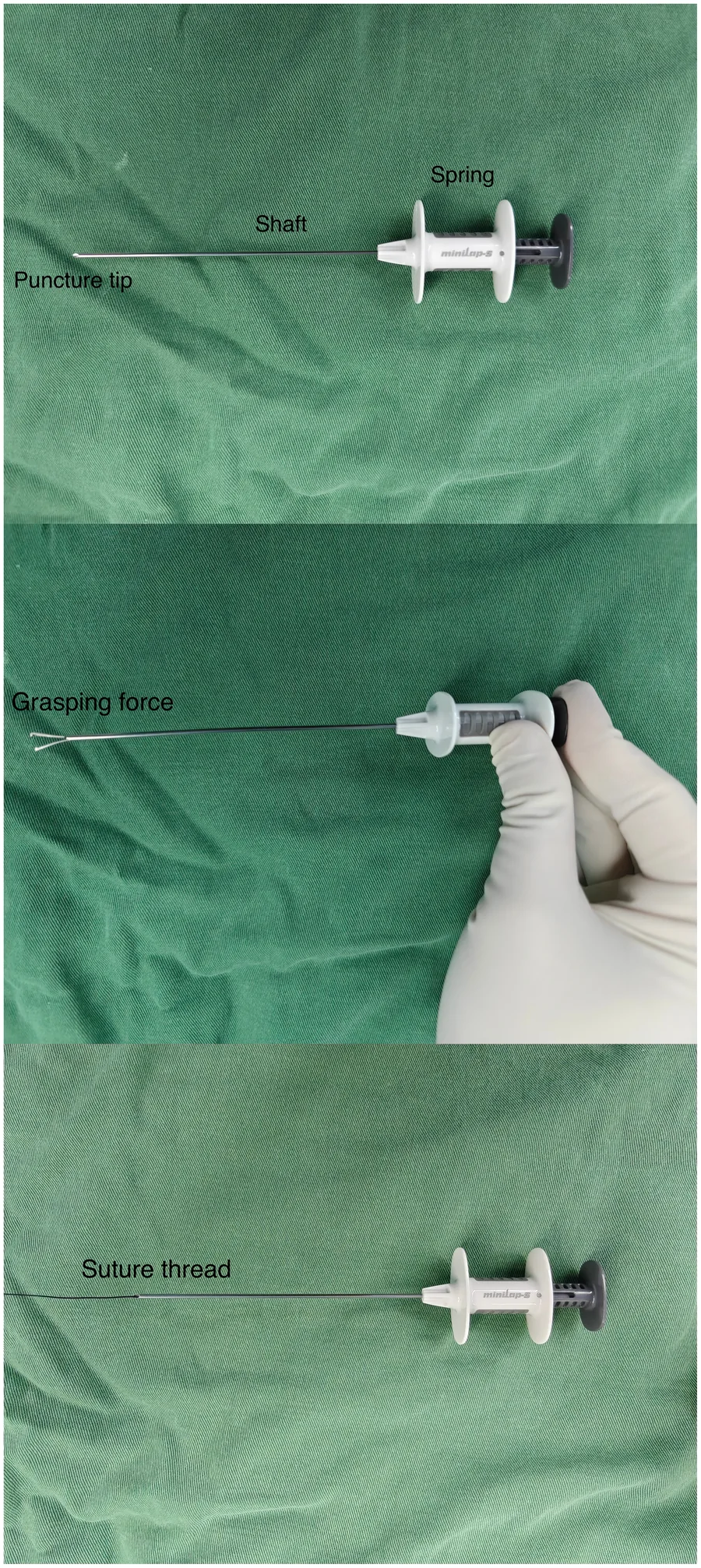
External appearance and operating procedure of an endo fascial closure device (when the spring at the end of the closure is pressed by surgeon, the grasping force extends from the hollow rod for grasping of the suture thread).

### Surgical technique

The patient was placed in a lateral decubitus position with the back elevated to 75° from the bed. A 5-mm trocar and a 5-mm thoracoscope were inserted through a transverse incision at the 5th intercostal space at the inferior angle of the scapula. Under thoracoscopic guidance, two 3-mm trocars were placed at the 5th intercostal space in the mid-axillary line and the 6th intercostal space in the anterior axillary line for instrumentation. The herniated viscera were reduced into the abdominal cavity by applying controlled pneumothorax pressure, thereby exposing the diaphragmatic defect. A 2-0 non-absorbable braided suture was pre-attached to the intended fixation point on the edge of the prosthetic patch (Figure 2). The patch was then introduced through the 5-mm port. To anchor the patch to the chest wall, the endo fascial closure device was introduced percutaneously through the chest wall at the intercostal space corresponding to the pre-placed suture, under thoracoscopic guidance. The spring-loaded grasping forceps was advanced to retrieve the suture end, which was then withdrawn extracorporeally through the device. The suture was tied directly to the periosteum of the rib, securing the patch to the chest wall (Figure 3). This maneuver was repeated at 2–3 additional points along the patch margin, spaced approximately 1 cm apart. The remaining circumference of the patch was then approximated to the native diaphragmatic rim using continuous suturing (2-0 non-absorbable) under slight tension. The patch covered the defect securely without tension or movement restriction. After confirming satisfactory repair, a chest drainage tube was placed in the ipsilateral pleural cavity. The incisions were closed in layers.

**Figure 2:**
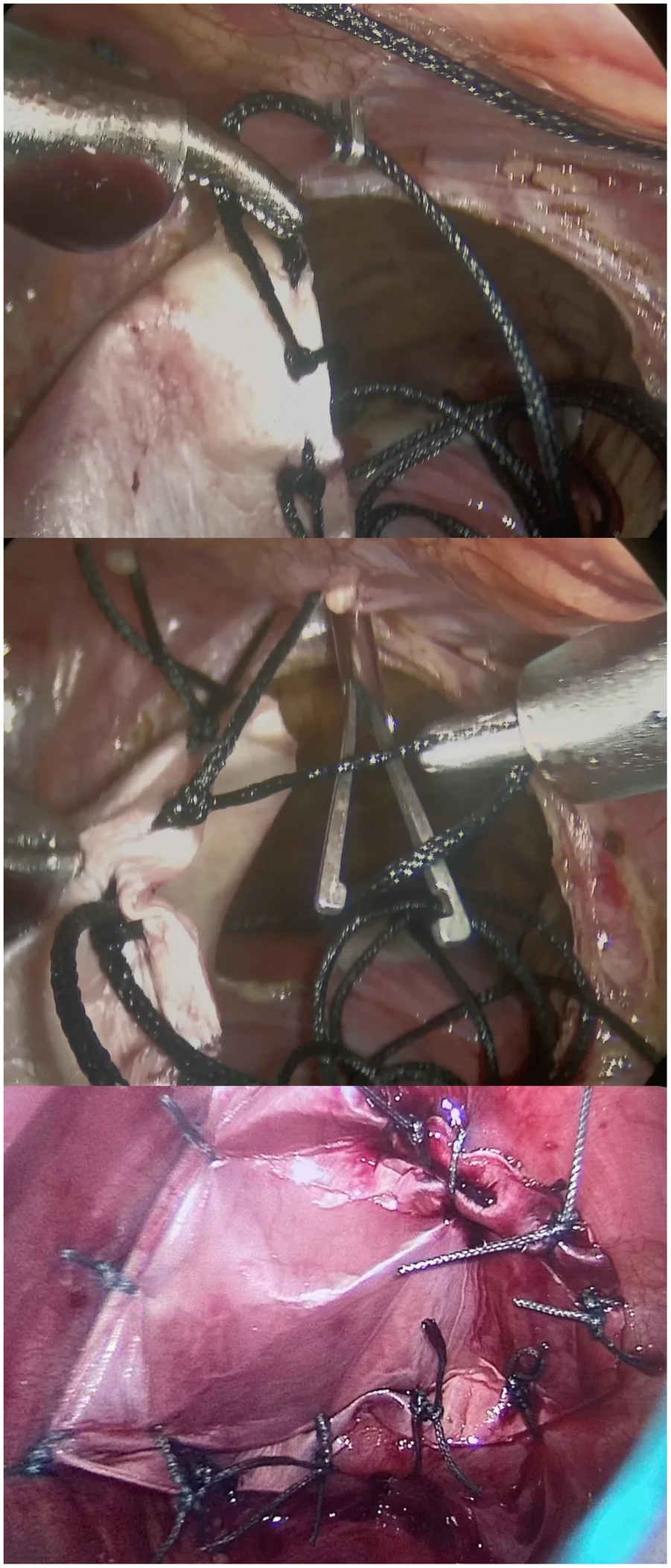
The puncture tip passes through the chest wall, grasps the pre-fixed silk sutures on both sides of the repair, and retrieves them out of the chest wall through the same puncture site.

**Figure 3:**
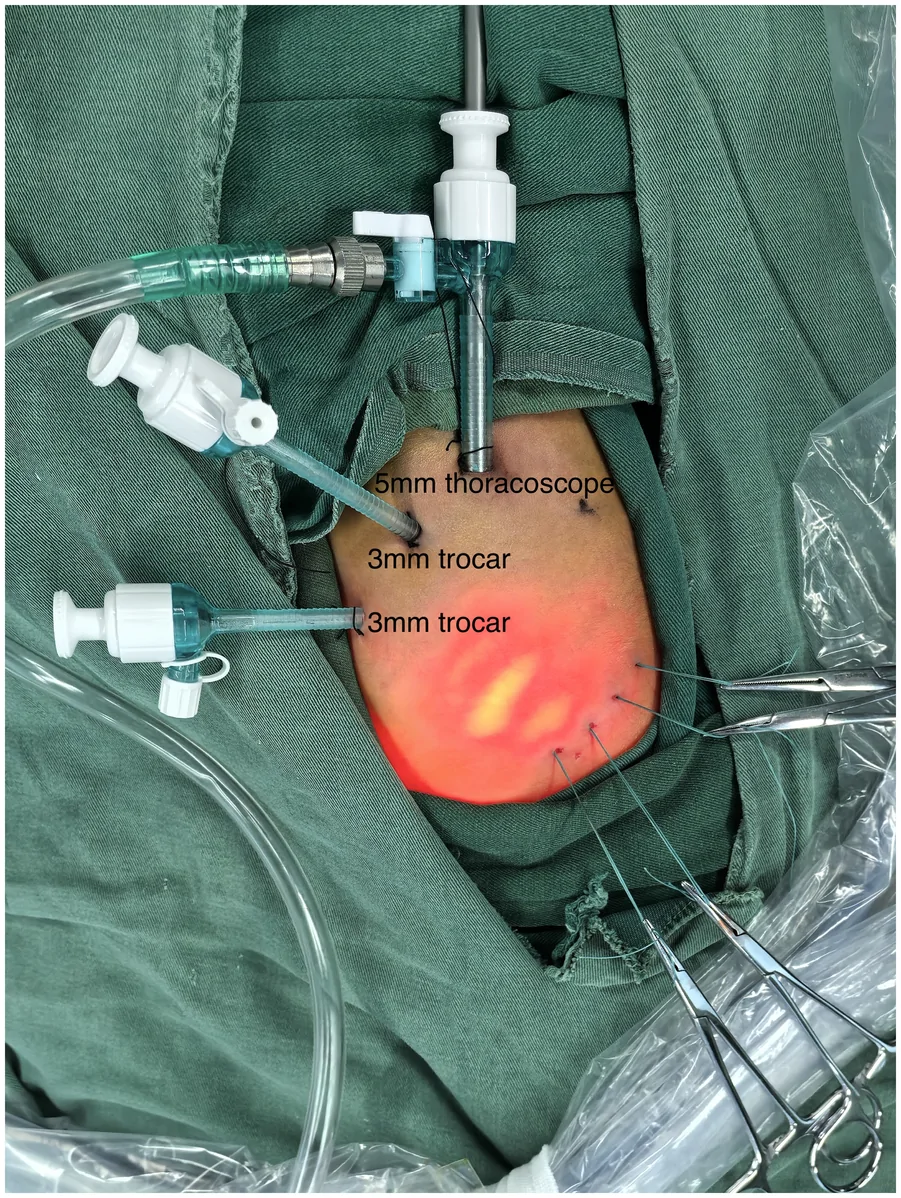
Extracorporeal schematic diagram (place the thoracoscope in 5 mm trocar, operating forceps and needle holder in 3 mm trocars, and fix the suture outside the chest wall with mosquito forceps).

To the neonates with CDH, CO2 pneumothorax pressure was established at 4–6 mmHg of pressure. A transient increase in insufflation pressure up to 8–10 mmHg was sometimes required to reduce the hernial contents. The high CO2 pneumothorax pressure (8–10 mm Hg) should last for only 2–3 min. All surgical procedures were performed by a single experienced pediatric surgeon, ensuring uniformity in operative technique, intraoperative decision-making regarding patch indication, and the application of the endo fascial closure device. This standardized approach minimized inter operator variability and allowed for a consistent evaluation of the described technique.

### Follow up

Postoperative follow-up was conducted according to a standardized protocol. All patients were evaluated at the following time points: 1 month, 3 months, 6 months, and every 6 months thereafter after discharge. At each visit, clinical assessment included growth parameters, feeding status, respiratory symptoms, and physical examination. Imaging surveillance consisted of chest radiography at 1 month, 6 months, and 12 months postoperatively, followed by annual chest radiographs thereafter. Upper gastrointestinal contrast studies were performed at 6 months postoperatively or earlier if feeding difficulties or symptoms suggestive of recurrence were present. Hernia recurrence was defined as the re-herniation of abdominal contents into the thoracic cavity confirmed by imaging. The median follow-up duration was 28 months (range: 11–59 months). The distribution of follow-up durations was as follows: 2 patients were followed for less than 12 months postoperatively, 7 patients for 12–28 months, and 9 patients for 28–59 months. Follow-up data were obtained through outpatient clinic visits or telephone/online interviews with the patients' families.

### Statistical analyses

All the collected data were statistically analyzed using SPSS 24.0 software. The continuous variables were expressed as the mean ± standard deviation, and categorical variables were expressed as frequencies (percentages).

## Results

We conducted thoracoscopic patch repair of congenital diaphragmatic hernia (CDH) using an endo fascial closure device in a cohort of 18 neonates (12 males and 6 females). The mean gestational age at birth was 38.4 weeks, with an average birth weight of 3,085 grams. All cases were Bochdalek hernias, comprising 5 left-sided and 13 right-sided defects. The median postnatal age at surgery was 2 days (range: 1–12 days), and the mean operative weight was 3,140 grams. Prenatal diagnosis was established in 12 patients, while the remaining cases were identified postnatally. Intraoperatively, a hernia sac was identified and confirmed in 10 patients. Given that the average large diaphragmatic defect measured 4.08 ± 1.57 centimeters in diameter (range: 2–8 cm), all cases required patch reinforcement. The mean operative duration was 141.2 ± 55.4 min (Table 1). Notably, the procedure was associated with minimal blood loss, averaging only 5 milliliters among all patients.

**Table 1:** Demographic data and clinical outcomes in patients (*n* = 18).

Demographics	
Male: Female (n)	12:6
Mean birth weight, g (± SD)	3,085 ± 466
Mean gestational week, week (± SD)	38.4 ± 1.21
Left-sided: Right-sided hernia (n)	5:13
Preoperative characteristics	
Median age, d (± SD)	2 ± 1.1
Mean weight, g ((± SD))	3,140 ± 439
Prenatal diagnosis, *n* (%)	12, 66.7%
Intraoperative data	
Hernia sac, *n* (%)	10, 55.6%
Mean diameter of hernia, cm (±SD)	4.08 ± 1.57 (range: 2–8)
Mean operation time, min (±SD)	141.2 ± 55.4
Mean blood loss, mL (±SD)	5 ± 1.31
Postoperative features	
Mean ventilator time, d (±SD)	8.39 ± 6.63
Mean length of postoperative hospital stay, d (±SD)	17.4 ± 9.73

All procedures were successfully completed thoracoscopically without conversion to open surgery, and there were no instances of solid organ laceration or gastrointestinal perforation. The mean duration of ventilator support was 8.39 days, while the average postoperative hospital stay was 17.4 days. Postoperative chest radiography revealed pneumothorax in 10 patients (55.6%) and pleural effusion in 12 patients (66.7%). Among the 10 patients with pneumothorax, all of them had small, asymptomatic pneumothoraxes that resolved spontaneously without intervention. Among the 12 patients with pleural effusion, all were mild and resolved with conservative management. The chest drainage tube was successfully removed at a mean of 8.1 ± 2.8 days postoperatively. Despite these complications, all surviving patients showed significant clinical improvement prior to discharge. Furthermore, no cases of surgical site infection, diaphragmatic eventration, or postoperative intestinal obstruction were documented. During a median follow-up period of 28 months (range: 11–59 months), no hernia recurrence was observed (Table 2). The overall survival rate in this series was 94.4% (17/18). The single postoperative mortality (5.6%) occurred in a patient who developed severe respiratory and hemodynamic compromise following surgery, requiring high-level postoperative extracorporeal membrane oxygenation (ECMO) support. Unfortunately, due to significant economic constraints, the family was unable to afford the ongoing costs of ECMO therapy and opted to withdraw further treatment, after which the patient succumbed. This case is included as a postoperative mortality in our analysis, as the need for ECMO reflects the severity of the underlying disease and the complexity of perioperative management, rather than a purely non-clinical outcome.

**Table 2:** Intraoperative and postoperative complications.

Intraoperative	
Conversion, *n*(%)	0
Solid organ laceration, *n*(%)	0
Gastrointestinal perforation, *n*(%)	0
Postoperative	
Pneumothorax, *n*(%)	10 (55.6%)
Pleural effusion, *n*(%)	12 (66.7%)
Wound infection, n(%)	0
Diaphragmatic eventration, *n*(%)	0
Intestinal obstruction, *n*(%)	0
Discharge	
Recurrence, *n*(%)	0
Survival, *n*(%)	17 (94.4%)

## Discussion

Thoracoscopic repair for CDH has progressively gained acceptance as a primary surgical approach over the past three decades, largely owing to its minimally invasive nature and associated benefits such as reduced postoperative pain, shorter recovery periods, and improved cosmetic outcomes (10). Despite these advantages, the utilization of patch repair in thoracoscopic CDH repair continues to present considerable technical difficulties, primarily attributable to the constraints of operating within a limited thoracic cavity and the often-extensive diaphragmatic defects encountered. These challenges frequently result in prolonged operative durations, which in turn elevate the risk of serious intra- and postoperative complications, including respiratory acidosis, pulmonary hypertension, and, in severe cases, mortality. Consequently, a central and persistent challenge in this field lies in developing surgical techniques that can effectively shorten operative time while ensuring secure patch placement, thereby reducing the likelihood of life-threatening complications such as respiratory acidosis and pulmonary arterial hypertension during thoracoscopic patch repair of diaphragmatic hernias. Several techniques have been used to accomplish the suturing of large defects in CDH cases, including Endo Close™ device (11), Nonabsorbable Spiral Tacks (12), Spiral Tacks (13) and so on. Here, we aimed to simplify the thoracoscopic patch repair of CDH by applying an endo fascial closure device.

Prolonged intraoperative CO2 insufflation increases the risk of hypercapnia and acidosis, posing a concern for thoracoscopic CDH repair. In a single-center study, the surgical time for patch repair was 194 min (148–260 min) (14). In the study using the Endo Close™ device, the surgical time was 197 min (100–343 min) (11). In contrast, our average surgical time was 141.2 ± 55.4 min, which appeared shorter in our series compared with previously reported historical data. The technology of endo fascial closure device has simplified the surgical process to reduce the operative time, which might potentially lower the incidence of postoperative hypercapnia in children.

Among several thoracoscopic studies, the average duration of postoperative mechanical ventilation ranges from 5.4 to 12.8 days (6, 15, 16), whereas the total hospital stay ranges from 18.2 to 44.5 days (1, 15, 17, 18). In our series, the average postoperative ventilation duration was 8.39 days, which is comparable to the reported range. However, the average hospital length of stay was 17.4 days—numerically shorter than previously documented values. Thus, although the duration of postoperative ventilatory support in our series falls within the range of prior reports, the reduced hospital stay suggests potential improvements in postoperative recovery efficiency.

In this study, postoperative pneumothorax occurred in 55.6% of patients, and pleural effusion in 66.7%. Both complications resolved rapidly during the early postoperative period, accompanied by corresponding symptomatic improvement, and the chest drainage tube was successfully removed within 8 days following surgery. A small residual pneumothorax or pleural effusion is clinically permissible and does not adversely affect postoperative recovery. There were no instances of conversion, solid organ laceration, gastrointestinal perforation, wound infection, diaphragmatic elevation, or intestinal obstruction in the present series. Compared with findings reported in other studies, the incidence of intraoperative and postoperative complications seemed to be lower than that reported in some historical series (14, 19).

Most studies have reported a higher recurrence rate associated with thoracoscopic surgery compared with open repair, ranging from 5% to 26.9% (16, 20, 21). When patch repair was required during thoracoscopic procedures, the recurrence rate increased to 16.7% and even as high as 40% (15, 16, 19, 22). Unlike previous reports of high recurrence rates after thoracoscopic surgery—particularly when patch repair was required—no postoperative recurrences were observed in our series. This finding suggests that the use of an endo fascial closure device suggests a potential reduction in the risk of recurrence, although this observation requires confirmation in a controlled study, warranting further investigation as a potential standard technique in thoracoscopic repair.

In two separate single-center studies, the postoperative mortality rates for thoracoscopic diaphragmatic hernia repair were 10.6% and 10% (23, 24), respectively, indicating comparable outcomes. In contrast, the mortality rate in our series was 5.6% (1/18), which is lower than those reported in the aforementioned studies. The sole death in our series occurred in a patient who required high-level extracorporeal membrane oxygenation (ECMO) support postoperatively; however, the family was unable to afford the associated costs, leading to withdrawal of treatment and death. The unusually high proportion of right-sided defects in this cohort (72.2%) may be attributed to referral bias or a true regional variation; further multicenter studies are warranted to confirm this observation.

Beyond the clinical outcomes reported above, we believe the primary contribution of this work is the description of a novel, simple, and reproducible surgical technique. First, the procedure is technically straightforward. The percutaneous puncture-and-retrieval maneuver eliminates the need for complex intracorporeal knot-tying or extensive dissection to expose the diaphragmatic rim for suturing. Second, the technique is adaptable to various defect configurations. Whether the defect is left- or right-sided, or whether the native diaphragmatic rim is partially present or largely absent, the device can be applied to anchor the patch to any available fixation point on the chest wall. In summary, we believe its simplicity represent meaningful contributions to the surgical armamentarium for CDH. These attributes may facilitate the wider adoption of thoracoscopic patch repair, particularly in centers where the technical challenges of the procedure have previously been a barrier.

In this report, an endo fascial closure device was used for patch fixation during the thoracoscopic repair of large diaphragmatic defects, allowing for easy, quick, and efficient anchoring of the patch to the edges of the defect or ribs. In conclusion, it is a simple and accessible technique that can facilitate thoracoscopic patch repair of CDH, especially in larger defects, without conversion. This technique could benefit surgeons by shortening surgical duration and reducing the difficulty of patch repair in successful thoracoscopic CDH. Using this simplified patch repair technique, we may finally be able to attain improved postoperative outcomes.

### Limitations

We acknowledge several important limitations in this study. First, this is a single-center retrospective case series without a concurrent control group. Although we compared our outcomes with historical data from the literature, these comparisons are subject to bias due to differences in patient selection, perioperative management, and institutional practices over time. Second, the relatively small sample size (*n* = 18) precludes multivariate analysis to adjust for potential confounders. Future prospective, randomized, or propensity-matched studies with larger sample sizes are warranted to rigorously validate the benefits of this technique over conventional approaches.

## Data Availability

The original contributions presented in the study are included in the article/Supplementary Material, further inquiries can be directed to the corresponding author.

## References

[1] Inoue M Uchida K Otake K Nagano Y Mori K Hashimoto K. Thoracoscopic repair of congenital diaphragmatic hernia with countermeasures against reported complications for safe outcomes comparable to laparotomy. Surg Endosc. (2016) 30(3):1014–9. 10.1007/s00464-015-4287-6 26092016

[2] Szavay PO Obermayr F Maas C Luenig H Blumenstock G Fuchs J. Perioperative outcome of patients with congenital diaphragmatic hernia undergoing open versus minimally invasive surgery. J Laparoendosc Adv Surg Tech A. (2012) 22(3):285–9. 10.1089/lap.2011.0356 22356206

[3] Kays DW Islam S Larson SD Perkins J Talbert JL. Long-term maturation of congenital diaphragmatic hernia treatment results: toward development of a severity-specific treatment algorithm. Ann Surg. (2013) 258(4):638–44; discussion 644-5. 10.1097/SLA.0b013e3182a53c49 23989050 PMC4690730

[4] Van Den Hout L Sluiter I Gischler S De Klein A Rottier R Ijsselstijn H. Can we improve outcome of congenital diaphragmatic hernia? Pediatr Surg Int. (2009) 25(9):733–43. 10.1007/s00383-009-2425-8 19669650 PMC2734260

[5] Tyson AF Sola R Jr Arnold MR Cosper GH Schulman AM. Thoracoscopic versus open congenital diaphragmatic hernia repair: single tertiary center review. J Laparoendosc Adv Surg Tech A. (2017) 27(11):1209–16. 10.1089/lap.2017.0298 28976813

[6] Costerus S Zahn K Van De Ven K Vlot J Wessel L Wijnen R. Thoracoscopic versus open repair of CDH in cardiovascular stable neonates. Surg Endosc. (2016) 30(7):2818–24. 10.1007/s00464-015-4560-8 26490767 PMC4912591

[7] He QM Zhong W Zhang H Li L Wang Z Tan Y. Standardized indications to assist in the safe thoracoscopic repair of congenital diaphragmatic hernia in neonates. J Laparoendosc Adv Surg Tech A. (2016) 26(5):399–403. 10.1089/lap.2015.0327 26989924

[8] Lacher M St Peter SD Laje P Harmon CM Ure B Kuebler JF. Thoracoscopic CDH repair–A survey on opinion and experience among IPEG members. J Laparoendosc Adv Surg Tech A. (2015) 25(11):954–7. 10.1089/lap.2015.0243 26488724

[9] Huang JS Lau CT Wong WY Tao Q Wong KK Tam PK. Thoracoscopic repair of congenital diaphragmatic hernia: two centres' Experience with 60 patients. Pediatr Surg Int. (2015) 31(2):191–5. 10.1007/s00383-014-3645-0 25430524

[10] Silen ML Canvasser DA Kurkchubasche AG Andrus CH Naunheim KS. Video-assisted thoracic surgical repair of a foramen of bochdalek hernia. Ann Thorac Surg. (1995) 60(2):448–50. 10.1016/0003-4975(95)00100-y 7646117

[11] Mansour S Curry J Blackburn S Mullassery D Thakkar H Ballington J. Minimal access surgery for congenital diaphragmatic hernia: surgical tricks to facilitate anchoring the patches to the ribs. Pediatr Surg Int. (2023) 39(1):135. 10.1007/s00383-022-05303-y 36805329 PMC9941218

[12] Poupalou A Vrancken C Vanderveken E Steyaert H. Use of nonabsorbable spiral tacks for mesh reinforcement in thoracoscopic repair of congenital diaphragmatic hernia. European J Pediatr Surg Rep. (2018) 06(1):e27–31. 10.1055/s-0037-1612618 PMC5864520 29577002

[13] A Riquelme M D Guajardo C A Juarez-Parra M A Elizondo R C Cortinas J. Thoracoscopic patch repair of congenital diaphragmatic hernia in a neonate using spiral tacks: a case report. J Neonatal Surg. (2015) 4(3):31. 10.47338/jns.v4.202 26290813 PMC4518188

[14] Shah NR Criss CN Burgi K Matusko N Geiger JD Perrone EE. Thoracoscopic patch repair of congenital diaphragmatic hernia: can smaller incisions treat larger defects? J Pediatr Surg. (2024) 59(6):1083–8. 10.1016/j.jpedsurg.2023.09.040 37867043

[15] Gander JW Fisher JC Gross ER Reichstein AR Cowles RA Aspelund G. Early recurrence of congenital diaphragmatic hernia is higher after thoracoscopic than open repair: a single institutional study. J Pediatr Surg. (2011) 46(7):1303–8. 10.1016/j.jpedsurg.2010.11.048 21763826 PMC4297678

[16] Zani A Lamas-Pinheiro R Paraboschi I King SK Wolinska J Zani-Ruttenstock E. Intraoperative acidosis and hypercapnia during thoracoscopic repair of congenital diaphragmatic hernia and esophageal atresia/tracheoesophageal fistula. Paediatr Anaesth. (2017) 27(8):841–8. 10.1111/pan.13178 28631351

[17] Fishman JR Blackburn SC Jones NJ Madden N De Caluwe D Haddad MJ. Does thoracoscopic congenital diaphragmatic hernia repair cause a significant intraoperative acidosis when compared to an open abdominal approach? J Pediatr Surg. (2011) 46(3):458–61. 10.1016/j.jpedsurg.2010.08.062 21376192

[18] Nam SH Cho MJ Kim DY Kim SC. Shifting from laparotomy to thoracoscopic repair of congenital diaphragmatic hernia in neonates: early experience. World J Surg. (2013) 37(11):2711–6. 10.1007/s00268-013-2189-0 23963346

[19] Criss CN Coughlin MA Matusko N Gadepalli SK. Outcomes for thoracoscopic versus open repair of small to moderate congenital diaphragmatic hernias. J Pediatr Surg. (2018) 53(4):635–9. 10.1016/j.jpedsurg.2017.09.010 29055487

[20] Gourlay DM Cassidy LD Sato TT Lal DR Arca MJ. Beyond feasibility: a comparison of newborns undergoing thoracoscopic and open repair of congenital diaphragmatic hernias. J Pediatr Surg. (2009) 44(9):1702–7. 10.1016/j.jpedsurg.2008.11.030 19735811

[21] Bishay M Giacomello L Retrosi G Thyoka M Garriboli M Brierley J. Hypercapnia and acidosis during open and thoracoscopic repair of congenital diaphragmatic hernia and esophageal atresia: results of a pilot randomized controlled trial. Ann Surg. (2013) 258(6):895–900. 10.1097/SLA.0b013e31828fab55 23604057

[22] McHoney M Giacomello L Nah SA De Coppi P Kiely EM Curry JI. Thoracoscopic repair of congenital diaphragmatic hernia: intraoperative ventilation and recurrence. J Pediatr Surg. (2010) 45(2):355–9. 10.1016/j.jpedsurg.2009.10.072 20152351

[23] Liem NT Nhat LQ Tuan TM Dung le A Ung NQ Dien TM. Thoracoscopic repair for congenital diaphragmatic hernia: experience with 139 cases. J Laparoendosc Adv Surg Tech A. (2011) 21(3):267–70. 10.1089/lap.2010.0106 21204646

[24] Wang H Liu X Wang H Hu Y Wu Y. Thoracoscopic repair of congenital diaphragmatic hernia in neonates: a report of 47 cases. Zhejiang Da Xue Xue Bao Yi Xue Ban. (2018) 47(3):283–8. 10.3785/j.issn.1008-9292.2018.06.11 30226330 PMC10393712

